# Engineering *Escherichia coli *for succinate production from hemicellulose via consolidated bioprocessing

**DOI:** 10.1186/1475-2859-11-37

**Published:** 2012-03-29

**Authors:** Zongbao Zheng, Tao Chen, Meina Zhao, Zhiwen Wang, Xueming Zhao

**Affiliations:** 1 Key Laboratory of Systems Bioengineering, Ministry of Education, Tianjin University, Tianjin 300072, People's Republic of China; 2 Department of Biochemical Engineering, School of Chemical Engineering and Technology, Tianjin University, Tianjin 300072, People's Republic of China

**Keywords:** Consolidated bioprocessing, *Escherichia coli*, Hemicellulose, Succinate, Xylan

## Abstract

**Background:**

The recalcitrant nature of hemicellulosic materials and the high cost in depolymerization are the primary obstacles preventing the use of xylan as feedstock for fuel and chemical production. Consolidated bioprocessing, incorporating enzyme-generating, biomass-degrading and bioproduct-producing capabilities into a single microorganism, could potentially avoid the cost of the dedicated enzyme generation in the process of xylan utilization. In this study, we engineered *Escherichia coli *strains capable of exporting three hemicellulases to the broth for the succinate production directly from beechwood xylan.

**Results:**

Xylanases were extracellular environment-directed by fusing with OsmY. Subsequently, twelve variant OsmY fused endoxylanase-xylosidase combinations were characterized and tested. The combination of XynC-A from *Fibrobacter succinogenes *S85 and XyloA from *Fusarium graminearum *which appeared to have optimal enzymatic properties was identified as the best choice for xylan hydrolysis (0.18 ± 0.01 g/l protein in the broth with endoxylanase activity of 12.14 ± 0.34 U/mg protein and xylosidase activity of 92 ± 3 mU/mg protein at 8 h after induction). Further improvements of hemicellulases secretion were investigated by *lpp *deletion, *dsbA *overexpression and expression level optimization. With co-expression of α-arabinofuranosidase, the engineered *E. coli *could hydrolyze beechwood xylan to pentose monosaccharides. The hemicellulolytic capacity was further integrated with a succinate-producing strain to demonstrate the production of succinate directly from xylan without externally supplied hydrolases and any other organic nutrient. The resulting *E. coli *Z6373 was able to produce 0.37 g/g succinate from xylan anaerobically equivalent to 76% of that from xylan acid hydrolysates.

**Conclusions:**

This report represents a promising step towards the goal of hemicellulosic chemical production. This engineered *E. coli *expressing and secreting three hemicellulases demonstrated a considerable succinate production on the released monosaccharides from xylan. The ability to use lower-cost crude feedstock will make biological succinate production more economically attractive.

## Background

Lignocellulosic biomass represents an abundant, low-cost and renewable source of fermentable sugars. It is an alternative candidate besides petroleum as feedstock for fuel and chemical production [1]. Generally, lignocellulosic biomass comprises of 35-50% cellulose, 20-35% hemicellulose and 10-25% lignin [2]. As the major component of hemicellulose, xylan is one of the most abundant natural polysaccharides with a β-(1, 4)-linked xylose homopolymeric backbone. The side groups can be substituted with arabinose, glucose, galactose or glucuronic acid, based on the sources of xylan [3]. Recent research on utilizing xylan as feedstock boost development of numerous-promising processes for a variety of fuels and chemicals, such as biodiesel [4], xylitol [5], biohydrogen [6] and ethanol [7,8]. However, high cost in depolymerization is a primary obstacle preventing the use of xylan as feedstock [9,10].

Currently, the conversion of xylan by microorganisms requires a two-step process: acid or enzymatic hydrolysis of xylan into monosaccharides followed by the bioconversion of pentoses into bioproducts [7]. Complete acid hydrolysis generates fermentation inhibitors like furfural and hydroxymethylfurfural that complicate biological utilization [11]. On the other hand, enzymatic hydrolysis usually employs xylanase preparations produced by organisms which featuring dedicated xylanase production with high cost and low efficiency [7]. An alternative approach, known as consolidated bioprocessing (CBP), could potentially avoid the cost of the dedicated enzyme generation step by incorporating enzyme-generating, biomass-degrading and bioproduct-producing capabilities into a single organism through genetic engineering [12].

*E. coli *has been characterized as a good xylose utilizer. However, it cannot directly hydrolyze xylan. The pathway of xylan hydrolysis should be incorporated to add saccharifying traits to the microorganism. Besides, nonpathogenic laboratory strains of *E. coli *generally excrete only trace amounts of proteins into the culture medium under normal growth conditions [13]. So additional engineering is necessary to enhance the protein secretion of the microorganism. Osmotically inducible protein Y (OsmY), one of the most efficient excreting fusion partners in *E. coli*, has been used as a carrier protein to excrete various recombinant proteins of different origin and sizes into the medium at high levels [14]. The conversion of xylan to biodiesel by *E. coli *has been successfully obtained in an aerobic fermentation with two OsmY and xylanases fusion proteins [4]. However, due to relatively low enzyme activity and the fermentation process selected, the hydrolysis and conversion efficiency was fairly low, and the fatty acid ethyl esters yield from xylan was less than a quarter of that from glucose.

In the process of xylan conversion, metabolic engineering and synthetic biology are emerging as powerful approaches to realize the biorefinery construction [15]. New cellular systems created could convert lignocellulose to a wide range of metabolites. One of these metabolites, succinate, has been identified as one of the top 12 building blocks from lignocellulosic biomass [16]. Succinate is widely used as diesel fuel additives, deicers, solvents, and detergent builders. Besides, its potential value, as a 4-carbon building block for a number of higher value polymers, also arouse great interests [17]. Currently, the majority of succinate is manufactured petrochemically from butane via maleic anhydride. To reduce the cost of the process, alternative low-cost renewable routes from biomass carbohydrates by microbial fermentation have been highly sought after [18]. *E. coli *strains which can efficiently ferment a variety of sugars presented as polymeric constituents of lignocellulose to succinate have been developed [19]. Fermentations of lignocellulosic hydrolysates to succinate by *E. coli *have been carried out using acid hydrolysates of rice straw [20], softwood [21] and enzymatic hydrolysates of corn stalk [22]. It is notable that these studies adopted separate hydrolysis and fermentation (SHF) that the process of lignocellulose hydrolysis was discrete with the process of succinate production. To our knowledge, succinate production process performed by *E. coli *from lignocellulose in the form of simultaneous saccharification and fermentation (SSF [9]) has not been demonstrated before, not to mention the CBP.

Herein a successful CBP of xylan to succinate performed by *E. coli *is described. The potential of hemicellulases production and secretion from *E. coli *were explored. Beechwood xylan was hydrolyzed by the engineering *E. coli*, and the monosaccharides produced were simultaneously converted to succinate with the same strain. It is believed that this is the first CBP designed for the conversion of xylan to succinate by metabolically engineered *E. coli *strains.

## Results and discussion

### Target gene selection

Endoxylanase and xylosidase were firstly chosen for xylan hydrolysis guided by the following considerations. Endoxylanase randomly cleaves the β-l, 4 bonds in the xylan backbone to yield oligosaccharides, xylobiose and xylose [23]. The resulting xylooligosaccharides are further attacked by xylosidase at the non-reducing end, generating xylose [2]. A significant synergy exists between the two enzymes in the process of xylan hydrolysis.

To explore suitable xylanases to be expressed and exported from *E. coli *for xylan hydrolysis, a list of hundreds of candidate enzymes on the basis of known activities with xylan or xylobiose as substrate was developed. These xylanases were prioritized according to the enzyme activity between 30-37°C which was the optimal temperature for *E. coli *growth and fermentation. As most xylanases exhibited maximum activities at high temperatures, the enzyme activities are relatively low between 30-37°C. Among them, seven candidate enzymes [24-29] with higher enzyme activities between 30-37°C were selected for the following operon construction and *in vitro *testing to screen the optimal hydrolase combinations in CBP, including three types of endoxylanases and four types of xylosidases which are listed in Table 1.

**Table 1:** Designations and sources of candidate xylanases

**Xylanase No**.	Gene name	Preferred substrate	Protein Size (kDa)	Source organism	GenBank ID	Reference
**E1**	*xylD*	xylan	45	*Fusarium graminearum*	NT_086561	[24]

**E2**	*xyn10B*	xylan	44.3	*Clostridium stercorarium*	2106153A	[25]

**E3**	*xynC-A*	xylan	28.2	*Fibrobacter succinogenes *S85	U01037	[26]

**X4**	*xsa*	xylobiose	38	*Bacteroides ovatus*	AAB08024	[27]

**X5**	*xynB*	xylobiose	56	*Bacillus pumilus *IPO	CAA29235	[28]

**X6**	*xsa*	xylobiose	61	*Selenomonas ruminantium *GA192	AAB97967	[29]

**X7**	*xyloA*	xylobiose	42	*Fusarium graminearum*	AAT84260	[24]

### *In vitro *xylanase characterization

To hydrolyze xylan with high efficiency, a great amount of endoxylanases and xylosidases must be simultaneously produced and exported into the broth by *E. coli *strains. In order to obtain the highest hydrolysis efficiency, twelve pA-E_m_X_n _series plasmids were transformed into *E. coli *BL21 (DE3), and all the 12 engineered strains expressing different endoxylanase-xylosidase combinations were tested to screen the optimal xylanases combination.

The enzyme activities of each xylanase combination *in vitro *are shown in Table 2. For all the 12 strains, the enodxylanase and xylosidase were co-expressed and secreted well, resulting in both endoxylanase and xylosidase activities in culture broth distinctly, whereas the host strain transformed with empty plasmid backbone did not exhibit any xylanase activity. The 12 xylanase expression plasmids were derived from the same parent plasmid, pTrc99z, with same copy number, same promoter driving xylanases expression, and same secretion mechanism adopted. However, the secretions of the proteins varied widely. This may be attributed to the different structures of the xylanases which would alter the efficiency of protein transportation and affect the secretion of other proteins. Among them, Z1170 secreted 0.95 ± 0.03 g/l protein at 24 h after induction with low cell density in shake-flask cultivation, showing great potential of *osmY *fusion strategy for extracellular recombinant protein production.

**Table 2:** The physiology and enzymatic properties of Z1EX0 series strains

Strain	Xylanases expressed	Max OD_600_	Protein secretion (g/l)		Endoxylanase activity		Xylosidase activity	
			
			8 h(16 h)^a^	24 h	(U/L)	(U/mg)	(U/L)	(mU/mg)
Bl21(DE3)	-	6.50 ± 0.17	0.03 ± 0	0.03 ± 0	n.d.^b^	n.d.^b^	n.d.^b^	n.d.^b^
Z1000	-	6.19 ± 0.08	0.04 ± 0	0.03 ± 0	n.d.^b^	n.d.^b^	n.d.^b^	n.d.^b^
Z1140	E_1_, X_4_	3.45 ± 0.04	0.30 ± 0.02	0.69 ± 0	398 ± 13	1.31 ± 0.07	24 ± 1.0	80 ± 4
Z1150	E_1_, X_5_	4.08 ± 0.12	0.19 ± 0.01	0.31 ± 0.03	65 ± 2	0.34 ± 0.01	14.3 ± 0.6	75 ± 4
Z1160	E_1_, X_6_	4.55 ± 0.05	0.10 ± 0.01	0.21 ± 0.02	75 ± 0	0.75 ± 0.04	6.1 ± 0.3	61 ± 5
Z1170	E_1_, X_7_	2.02 ± 0.05	0.60 ± 0.04	0.95 ± 0.03	420 ± 19	0.70 ± 0.04	46.3 ± 0.5	77 ± 3
Z1240	E_2_, X_4_	5.12 ± 0.12	0.10 ± 0.01	0.17 ± 0.01	274 ± 10	2.71 ± 0.19	8.0 ± 0.3	77 ± 6
Z1250	E_2_, X_5_	5.69 ± 0.29	0.11 ± 0.02	0.12 ± 0.03	50 ± 4	0.47 ± 0.03	6.8 ± 0.2	63 ± 7
Z1260	E_2_, X_6_	4.83 ± 0.05	0.07 ± 0	0.10 ± 0	90 ± 5	1.36 ± 0.04	4.5 ± 0.2	64 ± 1
Z1270	E_2_, X_7_	4.07 ± 0.05	0.34 ± 0.01	0.43 ± 0.02	423 ± 14	1.25 ± 0.04	29.3 ± 1.2	86 ± 3
Z1340	E_3_, X_4_	5.10 ± 0.03	0.08 ± 0.01	0.21 ± 0.01	1098 ± 28	13.22 ± 1.05	7.3 ± 0.2	88 ± 7
Z1350	E_3_, X_5_	3.97 ± 0.09	0.22 ± 0	0.51 ± 0.04	1512 ± 2	6.88 ± 0	10.9 ± 0.5	49 ± 1
Z1360	E_3_, X_6_	4.93 ± 0.10	0.08 ± 0	0.25 ± 0	996 ± 1	13.14 ± 0.01	5.9 ± 0.2	77 ± 1
Z1370	E_3_, X_7_	5.04 ± 0.05	0.18 ± 0.01	0.25 ± 0.01	2151 ± 14	12.14 ± 0.34	16.3 ± 0.2	92 ± 3

^a ^Except maximum OD_600_, the sampling time was at the late-exponential phase, which was at about 8 h after induction for strains Z13X0 and the two controls, and 16 h for the other strains.

^b ^n.d.: not detectable.

Among the seven strains with the maximum OD_600 _exceeding 4.5 tested in this study, Z1370 (coding endoxylanase XynC-A from *Fibrobacter succinogenes *S85 and xylosidase XyloA from *Fusarium graminearum*) exhibited the optimal enzymatic properties. Z1370 secreted 0.18 ± 0.01 g/l protein and exhibited a highest xylosidase activity of 92 ± 3 mU/mg protein, a relatively high endoxylanase activity of 12.14 ± 0.34 U/mg protein at 8 h after induction. It has been reported that xylosidase is the rate-limiting enzyme compared to endoxylanase in xylan hydrolysis [30], which is consistent with the results shown in Table 2. Considering the high xylanase activities especially xylosidase activity, the quantity of protein secretion as well as the effect of protein expression on cell growth of *E. coli*, the artifical operon P_trc_-E_3_X_7 _was chosen as the xylan hydrolases gene cassette in all the following host-development work.

### Effects of *lpp *deletion and *dsbA *overexpression on the secretion and activities of xylanases

In their previous works, Shin et al. (2007) demonstrated that with *lpp *gene (coding Braun's lipoprotein) deletion, the outer membrane permeability of *E. coli *was increased [31]. Periplasm-directed recombinant proteins could be secreted into the extracellular environment with high efficiency without significantly affecting cell growth [32]. To further enhance the secretion of the OsmY-fused xylanases which were extracellular environment-directed, *lpp *deletion of the engineering strains was explored.

The influences of *lpp *deletion on the secretion and activities of the xylanases are shown in Table 3. The *lpp *deletion strain Z1371 exhibited a higher ability to excrete proteins, resulting higher xylanases activities. However, the specific enzyme activity was lower than that of control strain Z1370 without *lpp *deletion. The cell growth was also significantly affected as the maximum OD_600 _was only 40% of Z1370, which is not consistent with Shin's report [32]. We have doubted that the probable reason for this distinction was the different media used for strain culture. In this work the medium was M9/1% glucose minimal medium, while Shin et al. (2007) used LB or M9/1.0% casamino acid medium instead. However when Z1371 was cultured in LB medium, this detrimental effect was not ameliorated (data not shown). The periplasmic leaky phenotype caused by *lpp *deletion is not specific. Besides xylanases, other periplasmic proteins could also escape from cells, which maybe the true reason of cell growth retardation and the specific enzyme activity reduction. On the other hand, if a co-culture which could distribute the metabolic burden by dividing the microorganisms into hydrolyzer and producer was conducted, the strain with *lpp *deletion would be a good candidate for polymers hydrolysis. In the co-culture system, the growth rate of hydrolyzer (strains with *lpp *deletion) was slower than the producer. As a result, most of the hydrolyzed sugars would be utilized by the producer. However, as to the single strain fermentation conducted CBP, *E. coli *with *lpp *deletion is not a good choice.

**Table 3:** The physiology and enzymatic properties of the engineering strains with *lpp *deletion or *dsbA *overexpression

Strain	Max. OD_600_	Protein (g/l)		Endoxylanase activity		Xylosidase activity	
		
		Intracellular	Extracellular	(U/L)	(U/mg)	(U/L)	(mU/mg)
Z1370	5.04 ± 0.05	2.16 ± 0.11	0.18 ± 0.01	2151 ± 14	12.14 ± 0.34	16.3 ± 0.2	92 ± 3

Z1371	2.09 ± 0.12	1.24 ± 0.07	0.41 ± 0.01	2919 ± 72	7.16 ± 0.17	31.8 ± 0.7	78 ± 2

Z2370	4.43 ± 0.06	1.77 ± 0.04	0.21 ± 0.01	3024 ± 97	14.44 ± 0.62	20.7 ± 0.1	99 ± 3

Except maximum OD_600_, the sampling time was at the late-exponential phase, which was at about 8 h for the three strains.

To facilitate disulfide bond formation in the periplasm, the *dsbA *gene encoding the disulfide oxidoreductase, E3 and X7 genes were co-expressed in strain Z2370. Xylanases excretion and the specific enzyme activities were slightly higher when the periplasmic oxidoreductase was co-expressed in *E. coli *as shown in Table 3. The increased excretion was probably due to the fact that DsbA co-expression increased the soluble protein level in the periplasm [14,33], which further improved the specific enzyme activities of xylanases.

### Optimization of the expression level of xylanases

**Figure 1:**
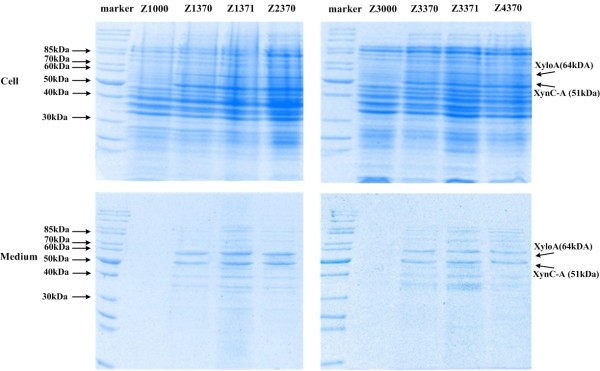
**Protein profiles of the engineering strains**. Cells were grown until OD600 reach 0.7 before IPTG was added to a final concentration of 0.5 mM, and incubation continued at 30°C. Samples were taken at 8 h after induction with IPTG. Proteins were separated on a 12% SDS-polyacrylamide gel.

In the course of transferring heterologous enzymatic pathways, it is easily induced a stress response from excessive heterologous protein by gene overexpression [34]. As shown in Figure 1, abundant xylanases were detected intracellular, which means that the capability of protein secretion was insufficient compared to that of protein expression. As aforementioned, two methods to assist xylanases export were investigated. However, even combination of expressing OsmY-fusion proteins in the *lpp *deletion strain, not all of the xylanases were taken out of the cytoplasm. It seems likely that this bottleneck is hardly to be removed. In order to coordinate the fluxes and avoid unnecessary waste, the upstream flux of the rate-limiting step should be reduced, which refers to protein production. In the shake-flask culture with M9/1% glucose medium, the maximum OD_600 _of BL21 (DE3) was 6.5 ± 0.17, and that of BL21 (DE3) harbouring pTrc99z plasmid was 6.19 ± 0.08. On the contrary, the maximum OD_600 _of Z1370 was only 5.04 ± 0.05, the maximum specific growth rate was also lower (data not shown). The main reason of the differences among these strains was the metabolic burden caused by plasmid-born and protein expression.

Plasmid copy number is an important factor in genetic engineering as it affects the protein expression level of the cloned gene by gene dosage effect and exerts a metabolic burden on the cell. The plasmids used in the above work were all pTrc99a-derivatived, that is, the origin of replication is pBR322 with copy number of 36-41 [35]. In order to alleviate the metabolic burden caused by plasmid conservation and protein hyperexpression, a new set of three strains including Z3370, Z3371 and Z4370 were engineered. The replication origins of the harboured plasmids were changed to p15A, the copy number of which were reduced to a relatively low number of 10-12 [36]. As seen from Table 4 the protein secretion of the new set of three strains were slightly lower than that of the corresponding strains harbouring pTrc99a-derivative plasmid, however, the maximum OD_600 _and specific growth rate were restored to a certain extent for these strains, especially for Z4370. This significant change can be attributed to the decrease of the cell's metabolic burden. The decrease of intracellular enzyme activities after changing the replication origins of the harboured plasmids also verified this conclusion to some extent from another side (data not shown). Though there were still a small amount of xylanases remained intracellular (Figure 1), the excess protein expression didn't severely hamper the cell growth anymore.

**Table 4:** The physiology and enzymatic properties of the engineering strains harboring a p15A-devirative plasmid

Strain	Max. OD_600_	Protein (g/l)		Endoxylanase activity		Xylosidase activity	
		
		Intracellular	Extracellular	(U/L)	(U/mg)	(U/L)	(mU/mg)
Z3370	5.49 ± 0.06	0.53 ± 0.03	0.16 ± 0.01	1945 ± 60	12.48 ± 0.57	15.0 ± 0.3	94 ± 4

Z3371	2.86 ± 0.05	0.13 ± 0.02	0.32 ± 0.01	2022 ± 16	6.32 ± 0.12	22.7 ± 0.3	71 ± 2

Z4370	5.75 ± 0.10	0.44 ± 0.02	0.18 ± 0.01	2581 ± 97	14.34 ± 0.67	17.0 ± 0.2	103 ± 3

Except maximum OD_600_, the sampling time was at the late-exponential phase, which was at about 8 h for the three strains.

Strains expressed other enzyme combinations including Z1170 and Z1350 were also tested. When the expression of xylanases reduced, the performance of the two strains were not as good as predicted. For strain Z1170, the maximum OD_600 _was restored from 2.02 to 2.95, however, the protein excretion was reduced to half. For strain Z1350, the maximum OD_600 _was unchanging, and the protein excretion was fell by a quarter.

### Effect of *abf2 *overexpression on the hydrolysis of xylan

**Figure 2:**
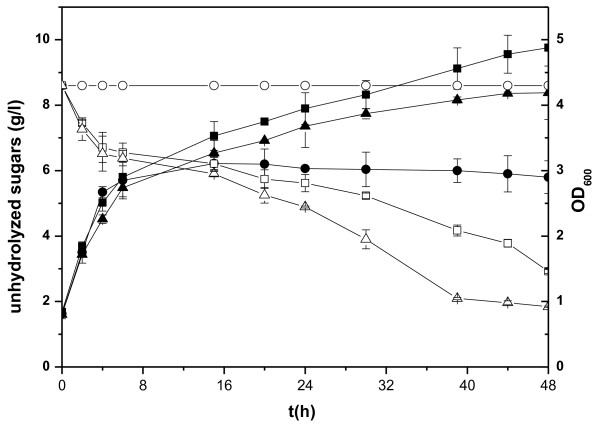
**Cell density and unhydrolyzed sugars during cultivation of the engineered strains**. Circle, Z3000; square, Z4370; triangle, Z5370; the solid symbols indicate OD_600 _and the hollow ones indicate unhydrolyzed sugars.

The biological activities of xylanases were further confirmed by using M9/0.2% glucose + 1% xylan minimal medium for cell growth. However, the enzymatic hydrolysis of xylan was insufficient compared to acid hydrolysis (Figure 2). After acid hydrolysis, 8.6 g reducing sugar was obtained from 10 g xylan while only 5.68 g was liberated in the process of enzymatic digestion, which was equivalent to 66% of acid hydrolysis reducing sugar. The uncompleted enzymatic hydrolysis may due to the lack of accessory hemicellulases [2]. The substitutions on the side chains of xylan may hinder the formation of enzyme-substrate complexes, thus impede enzymatic hydrolysis [37]. Arabinose is the most frequent substitution of natural hemicellulose [2]. The α-arabinofuranosidase catalyzes the hydrolysis of the non-reducing terminal α-arabinofuranosidic side chains which would physically restrict access to the β-(1, 4)-xylosidic linkage in the xylan backbone and slow the action of endoxylanase and xylosidase. The combination of xylanase, xylosidase, and arabinofuranosidase can completely hydrolyze arabinoxylan *in vitro *[25]. Therefore, pB-E_3_X_7_AD was constructed and transformed into BL21 (DE3) to obtain strain Z5370, in which *abf*2 (coding an α-arabinofuranosidase originated from *Bacillus subtilis *168 [38]), E3, X7 and *dsbA *genes were co-expressed. The effect of α-arabinofuranosidase on the improvement of hydrolysis efficiency of beechwood xylan is shown in Figure 2.

Forty-eight hours after induction, the degree of xylan hydrolysis was improved from 66% to 79% with α-arabinofuranosidase co-expressed (Figure 2), indicating the importance of removing this substitution for generation of monomeric sugars. At the end of cultivation, there were still a small amounts of oligomers of the enzymatic hydrolysates not taken up by *E. coli*, 9% for strain Z5370 while 15% for Z4370, which maybe substitutes of xylan that cannot be utilized by *E. coli *strains. It suggested that accessory hemicellulases should be existed in the broth to assist xylan hydrolysis. However, much more metabolic burden was exerted meantime, as shown in Figure 2. At 48 h after induction, the final OD_600 _of Z5370 was 4.2 while that of Z4370 was 4.9. A compromised strategy adopted should be concerned between the metabolic burden and the hydrolysis efficiency.

### CBP of xylan to succinate

Achieving a functional xylan hydrolysis pathway in *E. coli *was the first step in engineering an organism for CBP of xylan to succinate. The second major step involved optimizing the host strain to channel hydrolyzed monosaccharides into succinate production pathway. A classic double-knockout strategy was operated, removing lactate dehydrogenase (*ldhA*) and pyruvate formate lyase (*pflB*) genes to block the formation of byproducts (formate and lactate). In order to improve the xylose co-utilization efficiency with glucose, the glucose-specific permease of the phosphotransferase system (*ptsG*) gene was simultaneously knocked out to interfere with the regulation of catabolite repression [39]. In addition, *pyc *gene (coding a pyruvate carboxylase originated from *Corynebacterium glutamicum *ATCC 13032 [35]) was overexpressed to achieve higher yields of succinate by efficiently carboxylazing of pyruvate to oxaloacetate in this trimutant background.

**Figure 3:**
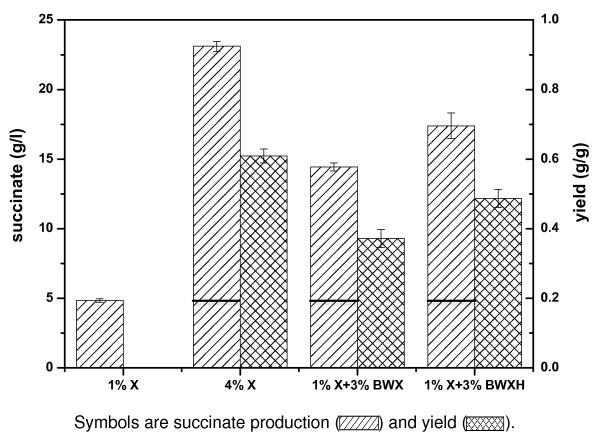
**Fermentation of Z6373 in different media**.

Xylan hydrolysis pathway was introduced into the succinate-producing strain above mentioned to give strain Z6373. Z6373 could directly convert xylan to succinate under oxygen-deprived conditions. Fermentation of xylan was carried out through a two-stage process in which cells harvested from aerobic fermentation were suspended in AM1/1% xylose (X) minimal medium supplemented 3% xylan (BWX) for CBP. Fermentations with 1% and 4% pure xylose as carbon source were also conducted for comparison. A summary of the results from these studies is listed in Figure 3. As an aerobic cultivation for two-hour induction in AM1 medium was conducted, the actual yield in anaerobic stage was hard to calculate accurately. In the following yield calculations, succinate produced from AM1/1% xylose (4.84 g/l) was subtracted. The differences were used to calculate succinate yields directly from carbon sources, including xylose, xylan and xylan acid hydrolysates.

A batch fermentation of Z6373 resulted in 14.44 g/l succinate at 120 h with 1% xylose and 3% xylan as substrates anaerobically, representing a yield of about 0.37 g/g xylan. Considering there were 9% hydrolyzed reducing sugars cannot be directly used by *E. coli*, the actual succinate yield would be higher. As shown in Figure 3, the yield from pure xylose was about 0.61 g/g xylose. The succinate yield from 10 g/l xylose was 4.84 g/l, so the succinate concentration of 14.44 g/l was a result of continued hydrolysis and fermentation of xylan during the anaerobic cultivation stage. To assess the extent of hydrolysis by hemicellulases in the fermentation process, acid hydrolysates (BWXH) were prepared and subsequently fermented by the strain. The succinate yield from acid hydrolysates was 0.49 g/g, 31% higher than that from xylan. The overall yield of succinate from beechwood xylan was lower than anticipated, which was not surprising. This is primarily due to incomplete xylan hydrolysis, as only three types of hemicellulases were produced. The consumption of carbon flux and energy flux for hemicellulases production and secretion was also a reason involved. The anaerobic productivities and yields in this study were somewhat lower than that reported in studies of producing succinate from lignocellulosic biomass [20,21]. However, those studies used complex medium, and the process of succinate production was discrete with the process of the lignocellulose hydrolysis, while in this work a CBP was conducted in minimal medium. We recognize that commercialization will require another two- to four-fold increase of production yield and much less fermentation time. However, the ability to directly use lower-cost crude feedstock will make biological succinate production more economically attractive, and feedstock flexibility will allow manufacturers to take advantage of geographic differences in sugar availability. Anyway, the final yield from xylan in this study was about 76% of that obtained from acid hydrolysates, indicating that significant hydrolysis was achieved by just co-expression of three hemicellulases.

To enable better expression of the xylanase genes, a microaerobic culture was tested. However, succinate produced under the microaerobic condition was much less than that under strict anaerobic condition (data not shown). This result was not unexpected, considering the traces of oxygen expected to supply for xylanase expression was preferentially diverted into the biomass formation flux, and most of the reducing sugars hydrolyzed by the xylanases were also changed to that channel. In microaerobic and aerobic cultures, a considerable part of carbon flux is used for cell growth which will be significantly affected the product output. This phenomenon is widespread in the conventional microaerobic or aerobic fermentation, which is much more serious for CBP as the monosaccharides supply is the bottleneck. In anaerobic fermentation, the strains were almost ceased to grow. Almost all the hydrolyzed sugars were converted into products. This indicated that anaerobic fermentation was more advantageous than microaerobic and aerobic fermentations with CBP of polysaccharides.

## Conclusions

This report represents a promising step towards the goal of hemicellulosic chemical production. In this work the production of succinate from hemicellulose was performed by *E. coli *strains via consolidated bioprocessing. Fusing with OsmY respectively, hemicellulases were secreted extracellularly with considerable enzyme activities. With three hemicellulases and a pyruvate carboxylase co-expressed, the engineered *E. coli *Z6373 was able to produce 0.37 g/g succinate from xylan anaerobically equivalent to 76% of that from xylan acid hydrolysates. This is the first example of culture designed and engineered specifically for CBP of xylan to succinate without using externally supplied hydrolases and any other organic nutrient. The ability to use lower-cost crude feedstock will make biological succinate production more economically attractive.

## Methods

### Strains, plasmids, and primers

*E. coli *strain TOP10 was used for propagation and amplification of plasmids; while *E. coli *strain BL21 (DE3) was employed for enzyme assays and characterizations and *E. coli *strain W1485 was engineering for succinate production. The features and descriptions of the used strains and plasmids in the present study are listed in Table 5. General recombinant DNA manipulations were performed by standard procedures [40]. All PCRs were carried out under the manufacturer's recommended conditions (Toyobo, Osaka, Japan). The primers used in the PCRs are listed in Table 6.

**Table 5:** Strains and plasmids used in this study

Strain	Genotype	Source
*E. coli *W1485	*F^+ ^rpoS396(Am) rph-1*	Lab stocked

*E. coli *BL21 (DE3)	*F' ompT gal dcm lon hsdSB(rB- mB-) λ(DE3 [lacI lacUV5-T7 gene 1ind1 sam7 nin5])*	Novagen

*E. coli *TOP10	*F' mcrA Δ(mrr-hsdRMS-mcrBC) Φ80lacZ ΔM15 ΔlacΧ74 recA1 araD139Δ(ara-leu) 7697 galU galK rpsL (Strr) endA1 nupG*	Invitrogen

Z0001	BL21 (DE3): Δ*lpp*	This study

Z0003	W1485: Δ*ldhA*, Δ*ptsG*::*cat*, Δ*pflB*::*kan*	This study

Z1000	BL21 (DE3): pTrc99z	This study

Z1EX0	BL21 (DE3): pA-E_m_X_n_	This study

Z1371	Z0001: pA-E_3_X_7_	This study

Z2370	BL21 (DE3): pA-E_3_X_7_D	This study

Z3000	BL21 (DE3): pTrc15a	This study

Z3370	BL21 (DE3): pB-E_3_X_7_	This study

Z3371	Z0001: pB-E_3_X_7_	This study

Z4370	BL21 (DE3): pB-E_3_X_7_D	This study

Z5370	BL21 (DE3): pB-E_3_X_7_AD	This study

Z6373	Z0003: pB-E_3_X_7_AYD	This study

**Plasmid**	**Description**	**Source**

pUC57-E_m_	pMB1 ori, *P_lac _: endoxylanase gene *(E_1_, E_2 _or E_3 _gene), Amp^r^	Genscript

pUC57-X_n_	pMB1 ori, *P_lac_: xylosidase gene *(X_4_, X_5_, X_6 _or X_7 _gene), Amp^r^	Genscript

pUC57-E_m_X_n _series	pMB1 ori, *P_lac_:osmY-endoxylanase *E_m_, *osmY-xylosidase *X_n_, Amp^r^	This study

pTrc99a	pBR322 ori, Amp^r^	Invitrogen

pTrc99z	pBR322 ori, Amp^r^	This study

pA-E_m_X_n _series	pBR322 ori, *P_trc_:osmY-endoxylanase *E_m_, *osmY-xylosidase *X_n_, Amp^r^	This study

pA-E_3_X_7_D	pBR322 ori, *P_trc_: osmY*-*xynC-A, osmY*-*xyloA; PdsbA: dsbA*, Amp^r^	This study

pACYC184	p15a ori, Cat^r ^Tet^r^	NEB

pTrc15a	p15a ori, Amp^r^	This study

pB-E_3_X_7_	p15a ori, *P_trc_: osmY-xynC-A, osmY-xyloA*, Amp^r^	This study

pB-E_3_X_7_D	p15a ori, *P_trc_: osmY-xynC-A, osmY-xyloA*; *PdsbA: dsbA*, Amp^r^	This study

pB-E_3_X_7_OD	p15a ori, *P_trc_: osmY-xynC-A, osmY-xyloA, osmY*; *PdsbA: dsbA*, Amp^r^	This study

pB-E_3_X_7_AD	p15a ori, *P_trc_: osmY-xynC-A, osmY-xyloA, osmY-abf2*; *PdsbA: dsbA*, Amp^r^	This study

pB-E_3_X_7_AYD	p15a ori, *P_trc_: osmY-xynC-A, osmY-xyloA, osmY*-*abf2*; *P_trc_: pyc*; *PdsbA: dsbA*, Amp^r^	This study

**Table 6:** Primers used in this study

Primer name	DNA sequence (5'-3')
osmY-f	GCGGAATTCATGAGATCTGGCTAACATAGGGTGGATCTATGACTATGACAAGACTGAAG

osmY-r	AATCTCGAGTTAGGATCCCCCGCTACCACTGCCCGAACCCTTAGTTTTCAGATCATTTT

99zD-f	ACATACCTCGAGCGCCTTTGAGTGAGCTGATA

99zD-r	CGACACTAGTTTGATCTTTTCTACGGGGTC

trc-f	GCAGCAATTGCGCGTATACGACAGCTTATCATCGACTG

trc-r	GTCGACTCTAGAGGATCC

dsb-f	GTACGAGCATATGTTCGACACCGCTGAAATCGG

dsb-r	AGCGCAGCATATGACGGCTAACGCAACAATAACACCT

184-f	GCATACTAGTGGCTTCCCGGTATCAACA

184-r	CTATCTCGAGCAGTACCGGCATAACCAAGC

abf2-f	GCCAGGATCCATGTCTGAACATCAAGCA

abf2-r	GCCATCTAGAGCATGACGTCTTAAGAATCAGCACGCAG

pyc-f	GCTGGAGCTCGACGCAATTGGGCTAACATAGGGTGGATCTATGTCGACTCACACATCTT

pyc-r	CTGCTCTAGATTAGGAAACGACGACGATC

Underlined nucleotides represent restriction sites.

### Operons and plasmids construction

The osmotically inducible protein Y gene (*osmY *[GenBank: CAQ34734]) was amplified from *E. coli *BL21 (DE3) genomic DNA using primers osmY-f and osmY-r, starting with a strong ribosome-binding sites and ending with a glycine-serine linker.

Three endoxylanase genes and four xylosidase genes (Table 1) were codon-optimized for enhanced expression in *E. coli*, and some restriction sites were added or eliminated to simplify subsequent manipulations according to the Bglbrick standard [41]. The seven codon-optimized genes were synthesized in the form of Bglbricks, and subsequently inserted into a modified pUC57 Bglbrick vector, respectively, resulting in three pUC57-E_m _(m = 1, 2 or 3) and four pUC57-X_n _(n = 4, 5, 6 or 7) plasmids (Genscript, Nanjing, China). According to the Bglbrick standard assembly, *osmY *was fused with different xylanase genes via the glycine-serine linker by inserting it into the seven pUC57-E_m _and pUC57-X_n _plasmids, respectively. The four resulting OsmY-xylosidase fusion genes were then recut and assembled with the other three plasmids containing OsmY-endoxylanase fusion gene to obtain twelve OsmY-endoxylanase-OsmY-xylosidase (E_m_X_n_)gene combinations presented in pUC57-E_m_X_n _plasmids, respectively.

A slightly modified medium-copy plasmid for the Bglbrick insertions, pTrc99z, was constructed by removing 14 bp ribosome-binding sites fragment downstream the *trc *promoter from pTrc99a. Twelve pA-E_m_X_n _plasmids were constructed by cloning the corresponding *EcoR*I-*BamH*I digested E_m_X_n _gene fragment from pUC57-E_m_X_n _on pTrc99z for *in vitro *characterizations of xylanases.

The disulfide isomerase I gene (*dsbA *[GenBank: ACT45539]) was amplified from *E. coli *BL21 (DE3) genomic DNA using primers Dsb-f and Dsb-r, digested with *Nde*I, and ligated into the same restriction site of pA-E_3_X_7_, resulting in pA-E_3_X_7_D.

Plasmids pTrc15a, pB-E_3_X_7 _and pB-E_3_X_7_D were constructed form pTrc99z, pA-E_3_X_7 _and pA-E_3_X_7_D respectively by replacing the pBR322 origin with p15a origin of pACYC184 using primers 99ZD-f, 99ZD-r, 184-f and 184-r.

The *osmY *gene digested with *Bgl*II-*BamH*I was ligated to the *BamH*I restriction sites of pB-E_3_X_7_D, resulting in pB-E_3_X_7_OD. The *abf*2 gene [GenBank: EU073712] which encodes an α-arabinofuranosidase amplified from *Bacillus subtilis *168 genomic DNA using primers abf2-f and abf2-r was cloned into the *BamH*I-*Sal*I sites of pB-E_3_X_7_OD to generate plasmid pB-E_3_X_7_AD.

A 300 bp fragment containing *trc *promoter was amplified from pTrc99z using primers trc-f and trc-r, digested with *Mfe*I and *Xba*I, and subsequently ligated into the *EcoR*I-*Xba*I sites of pUC18 to generate plasmid pUC18T. The pyruvate carboxylase gene (*pyc *[GenBank: CAA70739]) was amplified from *Corynebacterium glutamicum *ATCC 13032 chromosome using primers pyc-f and pyc-r with the GTG start codon changing to ATG, and inserted into the *Sac*I-*Xba*I sites of pUC18T to generate plasmid pUC18TY. Then the fragment containing *trc *promoter and *pyc *was cut with *Afe*I and *Spe*I from pUC18TY, and cloned into pB-E_3_X_7_AD at the *BstZ*171-*Xba*I sites to yield plasmid pB-E_3_X_7_AYD.

### Gene inactivation

The method used for the deletion of genes in the chromosome of *E. coli *including *lpp, ldhA, pflB *and *ptsG *was based on the utilization of λRed-mediated recombination [42]. If necessary, the FRT-flanked resistance cassette was removed from genome with the helper plasmid pCP20.

### Media and cultivation conditions

Engineering strains were cultured in LB, M9 [43], NBS or AM1 [44] medium as indicated. Appropriate antibiotics were added for plasmid maintenance or gene deletion (ampicillin at 100 μg/ml, kanamycin at 20 μg/ml or chloramphenicol at 5 μg/ml).

Xylanases characterization was operated in a 100 ml shake-flask cultivation using M9/1% glucose minimal medium. Protein expression was induced with 0.5 mM IPTG when the OD_600 _reached about 0.7. Simultaneously the growth temperature was switched from 37°C to 30°C with the agitation rate set at 200 rpm. Cells were incubated for an additional 16 h. Samples were taken periodically to monitor cell growth (OD_600_), protein, and reducing sugar concentration.

Xylan (xylose)-to-succinate conversion was detected in NBS and AM1 minimal medium. Cells were aerobically grown in a 250 ml Erlenmeyer flask containing 50 ml NBS/1% xylose medium with the temperature maintained at 37°C. When the OD_600 _reached 2.5, cells were harvested by centrifugation at 4000 g. Washed once with distilled water, the cells were suspended in a 250 ml Erlenmeyer flask containing 20 ml AM1 medium supplemented with 4% carbon source as described yielding an initial OD_600 _of approximately 6. Simultaneously, the cultures were induced with 0.5 mM IPTG. Two hours after induction, anaerobic cultivation was started by transferring the cultures into a 100 ml bottle sealed with a sterile stopper, while NaHCO_3 _and MgCO_3 _(basic) were added to a final concentration of 0.1 M each. Microaerobic conditions were also used for succinate production, which were established by piercing the stopper with a 16 G needle (Becton-Dickenson, San Jose, USA) after inoculation. The needle was kept in the bottle during growth to allow a small amount of air to enter. Cells were incubated anaerobically or microaerobically for an additional 120 h at 30°C with the agitation rate set at 200 rpm unless otherwise indicated in the text.

Acid hydrolysates of beechwood xylan were prepared as described [45]. The same fermentation conditions described above were used, except that the carbon source in AM1 medium was 1% xylose plus 3% (wt/vol) acid hydrolysates.

### Protein and enzyme analysis

Endoxylanase activities were determined using beechwood xylan as substrate in cell-free media by a modified method described [46]. The reducing sugar liberated in the reaction mixture was measured by the DNS method [47], and D-xylose was used as the standard. Xylosidase activities were measured using p-nitrophenyl-β-xylose (Sigma-Aldrich, St. Louis, USA) as substrate [24]. Those reactions were performed at 30°C for 40 min. One unit of enzyme activity was defined as the amount of enzyme that released 1 μmol of product (xylose or p-nitrophenol) equivalents in 1 min under the assay conditions.

Extracellular and intracellular protein fractions were prepared according to the method described in the pET system manual (EMD Chemicals, San Diego, USA). Protein concentration of each fractionated sample was determined as described by Bradford [48] with the commercial Bradford reagent (Sigma-Aldrich). SDS-PAGE was performed using 4% acrylamide stacking gel and 12% acrylamide resolving gel as described by Laemmli [49]. Protein bands were detected using Coomassie blue staining.

### Fermentation process analysis

Cell growth was monitored by measuring the optical density at 600 nm (OD_600_) with a UV-vis spectrophotometer (TU-1901, Persee, Beijing, China).

Fermentation products were analyzed by HPLC (HP1100, Agilent Technologies, Palo Alto, USA), using an ion exclusion Aminex HPX 87-H column (Bio-Rad, Richmond, USA) with 5 mM H_2_SO_4 _as the mobile phase at 0.6 ml/min flow rate, 55°C column temperature and UV absorption at 210 nm [19]. All samples for HPLC analysis were centrifuged at 13000 g for 10 min, and filtered through 0.2 μm filters before analysis.

## Competing interests

The authors declare that they have no competing interests.

## Authors' contributions

ZZ and MZ performed the experiments under the guidance of TC. ZZ analyzed the experimental data and drafted the manuscript. TC and ZW made substantial contributions to conception, interpretation of data and revised the manuscript. TC, XZ and ZZ developed the idea for the study and designed the research. All authors read and approved the final manuscript.
